# Providing a Placental Transfusion in Newborns Who Need Resuscitation

**DOI:** 10.3389/fped.2017.00001

**Published:** 2017-01-25

**Authors:** Anup C. Katheria, Melissa K. Brown, Wade Rich, Kathy Arnell

**Affiliations:** ^1^Neonatal Research Institute at Sharp Mary Birch Hospital for Women and Newborns, San Diego, CA, USA

**Keywords:** placental transfusion, delayed cord clamping, cord milking, hypovolemia, perfusion, resuscitation, blood volume, neonatal transition

## Abstract

Over the past decade, there have been several studies and reviews on the importance of providing a placental transfusion to the newborn. Allowing a placental transfusion to occur by delaying the clamping of the umbilical cord is an extremely effective method of enhancing arterial oxygen content, increasing cardiac output, and improving oxygen delivery. However, premature and term newborns who require resuscitation have impaired transitional hemodynamics and may warrant different methods to actively provide a placental transfusion while still allowing for resuscitation. In this review, we will provide evidence for providing a placental transfusion in these circumstances and methods for implementation. Several factors including cord clamping time, uterine contractions, umbilical blood flow, respirations, and gravity play an important role in determining placental transfusion volumes. Finally, while many practitioners agree that a placental transfusion is beneficial, it is not always straightforward to implement and can be performed using different methods, making this basic procedure important to discuss. We will review three placental transfusion techniques: delayed cord clamping, intact umbilical cord milking, and cut-umbilical cord milking. We will also review resuscitation with an intact cord and the evidence in term and preterm newborns supporting this practice. We will discuss perceived risks versus benefits of these procedures. Finally, we will provide key straightforward concepts and implementation strategies to ensure that placental-to-newborn transfusion can become routine practice at any institution.

## Introduction

The transition to extrauterine life is characterized by changes in circulation and initiation of ventilation and oxygenation via the lungs. Much of the focus surrounding neonatal resuscitation has historically been on the ventilation and oxygenation of the transitioning infant, including methods of providing ventilator support and determining how much oxygen is appropriate. Placental transfusion has only recently been recognized as an important factor in this fetal/neonatal transition. Allowing a natural transition by not clamping the cord until it has stopped pulsing was the norm for centuries until the 1950s when concern over maternal anesthesia crossing the placenta to the infant caused Virginia Apgar to suggest immediate clamping of the umbilical cord. In 1962, a few years after this recommendation, data were available showing that clamping of the cord immediately after birth yielded a marked bradycardia in term infants, but practice did not change (1).

## Physiology of Placental Transfusion

The goal of placental transfusion is to facilitate the transfer of blood volume from the placenta to the newborn. Owing to the relatively large size of the placenta compared with the fetus at mid-term, blood is equally distributed between the fetus and placenta. By term gestation, about one-third of the blood flows through the placenta and two-thirds flows through the fetus at any point in time (1). It follows that immediate cord clamping (ICC) results in one-third of total blood volume remaining in the placenta. Delaying clamping of the cord (DCC) for 60 s decreases the residual placental blood to 20% and by 3–5 min the residual placental volume is approximately 13% (2). When the umbilical cord is clamped, the low-resistance vascular bed of the placenta is disconnected, leading to an immediate increase in the newborn’s systemic vascular resistance. In a study on anesthetized fetal lambs, Bhatt et al. demonstrated a 50% drop in pulmonary blood flow and an abrupt 40% drop in heart rate (due to cessation of umbilical venous flow from the placenta) in anesthetized fetal lambs receiving ICC (3).

### Positioning of Infant

Yao and Lind found that gravity affects the amount of placental transfusion. Holding the neonate high above the placenta (head 40–60 cm above) decreases placental transfusion similar to ICC (4). A recent study found no difference in infant weights after DCC for 2 min with infants placed on the maternal abdomen versus at the introitus (5). However, total weight gain was half of what was previously found (6, 7), indicating that 2 min may not be enough time for a full placental transfusion for the term infant. Mercer and Erickson-Owens found that term infants placed on the maternal abdomen immediately after birth who were assigned to DCC for 5 min received a significantly larger placental transfusion than those with a 2-min delay (8), and more recently found that term infants placed skin to skin had significantly less residual placental blood volume than infants with immediate clamping (9).

## Methods of Providing a Placental Transfusion

### Delayed Cord Clamping in Preterm Infants

International Liaison Committee on Resuscitation (ILCOR) and other organizations recommend a 30- to 60-s delay in cord clamping during preterm birth (10, 11). Several randomized controlled trials and meta-analyses have been published on DCC in premature newborns (12–27). Although DCC decreases the *overall* incidence of intraventricular hemorrhage (IVH), enthusiasm for DCC is tempered by the lack of benefit for severe IVH and/or death in addition to the small numbers of newborns included in these trials and concerns about reporting bias (28). Recently, the largest DCC trial (*n* = 208) did not show any difference in severe IVH (9). The lack of benefit could reflect the inadequate placental transfusion during DCC for newborns delivered by cesarean section (C/S). Three trials of DCC versus ICC stratified by mode of delivery found no difference in hematocrit levels or tagged red blood cells in newborns delivered by C/S (29–31). ACOG acknowledges that there are limited data indicating whether DCC performed during C/S can improve placental transfusion (32). Various trials have had significant protocol violations, with 14–22% of newborns randomized to DCC actually receiving ICC (9, 29). It has been suggested that high pulmonary arterial pressure may be protective against fluctuating cerebral blood flow by reducing ductal shunt in extremely preterm infants (33). We speculate that improved cerebral blood flow, oxygen carrying capacity, increased pulmonary arterial pressure, and reduced ductal shunt following placental transfusion may contribute to reduced IVH observed in preterm infants following DCC (28).

### Delayed Cord Clamping in Preterm Infants Who Require Resuscitation

It has been suggested that cord clamping should not occur until the newborn is breathing (3, 34), as this may result in an inadequate transfusion in depressed infants who are not breathing during the delay (35). Nevill and Meyer compared non-breathing with breathing newborns who received DCC and found that non-breathing infants had a lower 1-min Apgar score, were more likely to be intubated, and develop chronic lung disease or severe IVH (35). In a related animal study, Polglase et al. (36) demonstrated improved cerebral and systemic oxygenation in preterm lambs if ventilation was provided before cord clamping.

However, it is unclear in preterm newborns whether a few gasping breaths or positive pressure ventilation is required. In our feasibility trial in which infants were randomized to receive ventilation during DCC or DCC alone showed no difference between provision of early positive pressure (by CPAP/positive pressure ventilation) or gentle tactile stimulation with bulb suctioning for 60 s during DCC. In videotaped deliveries, the incidence of infants who failed to respond to stimulation and remained apneic during DCC in the control arm and intervention arm was 5 versus 3%, respectively (*p* = 0.67). The time until the first breath in the control and intervention arm were 23 ± 19 and 23 ± 20 s in each arm, respectively (*p* = 0.93).

### Delayed Cord Clamping in Term Infants

International Liaison Committee on Resuscitation and other organizations recommend DCC for longer than 30 s in term infants (11, 13, 32). There is great variation in the timing of “late” cord clamping ranging from 30 s to 5 min to when the cord stops pulsating. There does not appear to be evidence that delay cord clamping in term newborns has a significant effect on neonatal death or morbidity outcomes such as admission to the neonatal intensive care unit or Apgar score less than 7 at 5 min (7, 11). Historically, studies have been limited to subjects who did not require resuscitation due to the perceived complexity of providing supportive interventions with the umbilical cord intact. In a comprehensive review of term infants receiving DCC (7), in 12 of the trials evaluated including 3,139 infants, mean birth weight was significantly higher in late clamping as opposed to early clamping. In 884 infants evaluated for hemoglobin (Hgb) concentrations, DCC babies had higher Hgb at 24–48 h of age. Four trials that evaluated term infants at 3–6 months of age who received DCC found a mean increase in serum ferritin with late clamping (37–40). There was no increased risk of polycythemia [hematocrit (Hct) >65%] in three trials that reported on the risk with DCC (37, 41, 42), although Ceriani Cernadas did find that the 3-min delay group had a significantly higher prevalence (14.1%) compared to immediate clamping, which was not found with the 1 min delay group (5.9%).

### Delayed Cord Clamping in Term Newborns Who Require Resuscitation

Approximately 10–15% of the babies born each year will need some sort of resuscitation at birth to successfully transition to extrauterine life. Positive pressure ventilation will be utilized in approximately 3–7% of newborns with 2% of infants requiring intubation and 0.1% needing chest compressions and/or epinephrine. In 85% of babies born at term gestation, spontaneous respirations will occur within 10–30 s of birth (10, 34, 43). Many have suggested that clamping the umbilical cord should be delayed in all births because of the associated benefits of increased blood volume, reduced need for blood transfusion, and iron deficiency (13, 15, 28, 38, 44–46). The American Academy of Pediatrics and the ILCOR do not yet recommend cord clamping for newborns who require resuscitation at birth due to limited evidence of benefit in this population (10, 47).

Neonates who are depressed at birth and require some resuscitation are the most likely to benefit from continued placental gas exchange and a placental transfusion with DCC. Beneficial cardiovascular changes can occur during DCC as pulmonary blood flow is established as a replacement for preload to the left heart before removing the placenta from the circulation. Maintaining venous return and cardiac output by avoiding ICC may help avoid adding a hypovolemic ischemic insult to an already asphyxiated infant (48). In a study of 20 healthy term newborns with 5 min of DCC, an improvement in stroke volume and cardiac output was demonstrated over the first 5 min of life. The mean time to cord pulsation cessation was 199 ± 76 s but cardiovascular benefits extended beyond the time of cessation (49). The term infant with shoulder dystocia whose umbilical cord is constricted as the neonate is squeezed in the birth canal is likely to have a portion of his blood shunted to the placenta (50, 51). The distressed newborn with a tight nuchal cord that is cut before birth may have up to 60 ml of their blood volume to remain in the placenta. Both scenarios may lead the infant to experience hypovolemia at birth (52). Hypovolemia at birth contributes to a poor transition and immediately clamping the umbilical cord can add additional cardiovascular complications with severe hypovolemia shock leading to asystole, an inflammatory cascade, hypoxic ischemic encephalopathy, seizures, and death (51, 53). The establishment of ventilation before clamping of the umbilical cord facilitates the cardiovascular changes of transition from fetal circulation and facilitates gas exchange in the lung, which is critical for adequate oxygenation of the newborn (48).

### Umbilical Cord Milking (UCM)

For the purposes of this review, we will delineate UCM as intact cord milking as compared to a second less studies method of cutting the umbilical cord and milking it one to three times. Currently, neither form of cord milking is recommended by ILCOR. ILCOR states, “In light of the limited information regarding the safety of rapid changes in blood volume for extremely preterm infants, we suggest against the routine use of cord milking for infants born at less than 29 weeks of gestation outside of a research setting. In making this recommendation, we place a higher value on the unknown safety profile and less value on the simplicity/economy of this intervention.” We will review the current evidence on cord milking in term and preterm infants.

### Cut-Umbilical Cord Milking (C-UCM)

Cut-umbilical cord milking is another technique, whereas the cord is immediately cut approximately 25 cm from the umbilicus and the baby is passed to the pediatric team and placed on the radiant warmer. The umbilical cord is raised and milked from the cut end emptying all of the contents into the baby. Upadhyay et al. studied 200 infants >35 weeks, half received C-UCM and half received early cord clamping (within 30 s) (54). The mean Hgb and Hct of the C-UCM group was significantly higher (*p* = 0.0001) at 12 h of age. The mean blood pressure was significantly higher at 30 min, 12 h, and 48 h after birth although within normal limits. Mean Hgb and mean serum ferritin were significantly higher in the C-UCM group at 6 weeks of age. There was no significant difference in polycythemia, serum bilirubin, and need of phototherapy between groups. Jaiswal et al. compared C-UCM to DCC of 60–90 s (100 in each group) in babies born >36 weeks gestational age (55). Mean serum ferritin at 6 weeks of age in the C-UCM group (134.0 ± 89.8 ng/ml) was comparable to DCC (142.7 ± 87.1 ng/ml). Mean Hgb at 6 weeks of age was also comparable 11.0 ± 2.4 g/dl in the C-UCM group and 11.3 ± 2.6 in the DCC group. There is the potential for C-UCM to affect the cerebral flow dynamics. In a substudy, which is the first to look at cerebral flow dynamics in term infants, Jaiswal et al. measured resistive index, pulsatility index, and cerebral blood flow velocities of middle cerebral artery at one point between 24 and 48 h of life by cranial ultrasound. The cerebral blood velocities and cranial Doppler indices were similar in C-UCM and DCC. The limitations of this study are a lack of sequential measurements and the anterior cerebral artery was not evaluated.

### Cord Milking in Term Infants

The mechanism in UCM is different from the passive transfusion that occurs at slow rate with DCC and which usually relies on the contraction of the uterus. UCM can be done rapidly and does not depend on uterine contraction and may be desirable in the situation of a depressed infant who requires resuscitation. In 1954, Colozzi studied 100 term infants who had their umbilical cord milked five times as compared to ICC and found improved Hgb and Hct levels and no adverse effects (56). Erickson-Owens et al. studied 24 term infants born by C/S and found improved Hgb and Hct levels at 36–48 h compared to ICC (57). In the ICC group, five infants had a capillary Hct level <47% suggestive of anemia. There was no report of symptomatic polycythemia, hyperbilirubinemia requiring hospitalization, or readmission for phototherapy.

### UCM and Resuscitation

Cord milking may offer an advantage over DCC in newborns who are deemed too unstable to wait for 30–60 s required for DCC. UCM can be performed in any low-resource setting and provides adequate placental transfusion without delay. Milking can be done in 15–20 s, equivalent to the time it takes for ICC (58). Critics of UCM cite that cord milking may occur before the establishment of respirations and pulmonary blood flow (48). In fact, UCM improves onset of respirations and pulmonary blood flow immediately at birth. This has been shown with recordings of electrocardiographic changes; newborns who had cord milking had a longer P wave, PR, and QTC interval when compared with those who had early clamping of the cord (59). Jaykka demonstrated that alveolar patency occurs in response to the filling of the surrounding capillaries, which may accelerate onset of respiration (60). This could explain why UCM may enhance earlier onset of breathing compared to DCC. In a study comparing DCC to UCM, more infants were breathing by the time the cord was clamped with UCM compared to a 45-s DCC (74 versus 53%) (29). This study provided evidence that UCM may be superior, and a larger multicenter trial is being planned. Our prior work showed that UCM increased heart rate and oxygen saturation within the first 5 min of birth, suggesting optimal transition compared to ICC. We also found that UCM decreased the number of days on oxygen therapy and reduced chronic lung disease.

### UCM in Preterm Infants

Umbilical cord milking has also been shown to have a positive impact on heart rate and other physiologic and hemodynamic measures when compared to ICC in preterm infants (61). Katheria et al. conducted a randomized controlled trial of 60 infants less than 32 weeks gestation randomized to UCM (20 cm stripped three times) or ICC to determine if there were differences in early physiology. They found that in the first 10 min of life, infants receiving UCM had higher heart rates, higher oxygen saturation, and were given less supplemental oxygen (all *p* < 0.05) when compared to infants receiving ICC (58). In a trial of 154 preterm infants, Katheria et al. found that UCM was more effective than DCC in preterm infants delivered by C/S as evidenced in improved blood flow (measured by echocardiography), blood pressure, renal perfusion (measured by urine output), and initial body temperature (29). Prior work has demonstrated that UCM increased heart rate and oxygen saturation within the first 5 min of birth suggesting optimal transition compared to ICC (62). UCM decreases the number of days on oxygen therapy and reduced chronic lung disease, which may be related to enhanced pulmonary blood flow at birth.

## How to Implement Resuscitation During Delayed Cord Clamping

In the hospital setting providing for resuscitation at the mother’s bedside with the umbilical cord intact creates some challenges that require careful planning, cooperation, and communication between the mother’s and the neonate’s health care teams. Specialized portable equipment designed to provide all the necessary tools for a safe and effective resuscitation must be available and the users must be familiar and proficient with its function (63). The labor and delivery suites and the operating theater have unique environmental considerations, space limitations, and requirements for sterility. Performing the resuscitation of a critically ill neonate so close to the patient’s mother can create stress on the medical personnel and family alike.

### Planning and Cooperation

Limited space and competing interests in the care of the mother and child dictate that a careful plan must be made in advance with the obstetrician (OB), mother’s nurse, and the neonatal team. The neonatal team’s close proximity to the mother while caring for the infant can interfere with the ability of the mother’s nurse and the OB to have access to care for the mother in the way they are accustomed. A plan must be devised ahead of the birth for stationing of equipment and personnel to avoid safety hazards and interference with the normal work flow. There should be the pre-assignment of roles in the care and the timing of interventions. The Apgar timer can be used to keep track of the time elapsed since birth, and the team should monitor and clearly announce the time every 15 s to facilitate the clamping of the cord at the desired interval. In the labor and delivery suites where more family and staff are present, one team member should be assigned to crowd control to oversee and assure an appropriate space is made for the family and critical staff. Simple tools that may usually be available during resuscitation such as the Apgar timer or newborn oxygen saturation goals must still be accessible and within view in the new arrangement.

### Specialized Equipment

**Figure 1:**
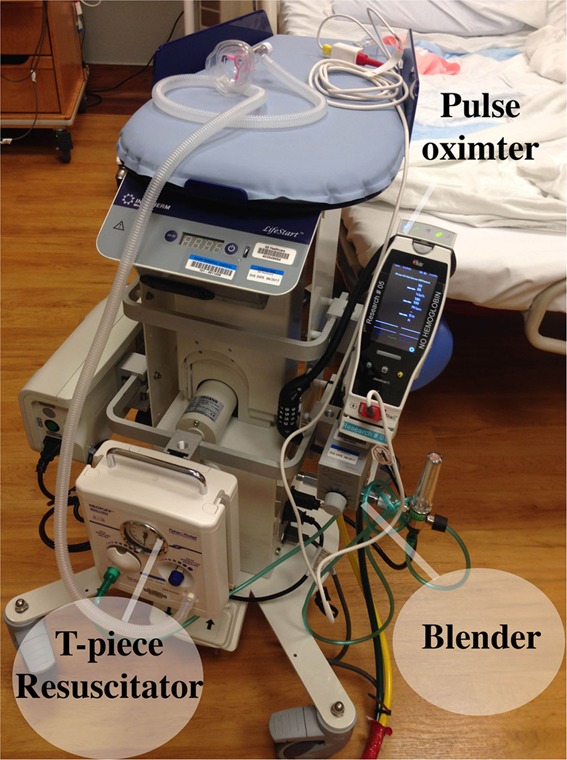
**Lifestart™ bed equipped with resuscitation equipment**.

Careful preparation is required to assure that equipment that might be needed to perform a successful neonatal resuscitation with an intact umbilical cord is available and is functional. The Lifestart™ bed (Inditherm Medical, Rotherham, UK) has been used by ourselves and others and is designed to provide a safe, adjustable, platform for the infant with space provided for the mounting of equipment (64, 65). We outfitted our Lifestart™ beds with a T-piece resuscitator, an air–oxygen blender, an oxygen flowmeter, and 50 psi air and oxygen hoses to connect to gas supply in any environment (Figure 1). Air and oxygen tanks can also be utilized, but have the disadvantage of being heavy, require checking for adequate contents and need replacement, and they can also be inadvertently left on creating a situation of inadequate gas supply. In addition, we mounted a pulse oximeter and a bag to hold disposable supplies such as a water proof cover for the stand, a ventilation circuit, resuscitation mask, oximeter probe, bulb suction, and enough extension tubing to connect to the wall suction if needed. The two gas supply hoses and the electrical cord for the Lifestart™ bed were lashed together to form one to improve the safety and maneuverability of the trolley.

### Considerations for Cesarean and Vaginal Deliveries

Mode of delivery may have a significant effect on placental transfusion. It has been speculated that perhaps more blood remains in the placenta when a neonate is delivered by C/S because the anesthetic and surgical interventions interfere with the active contraction of the uterine muscles to expel the placenta. Rabe et al. randomly assigned 58 neonates born at <33 weeks’ gestation to UCM (four times) or to a 30-s delay in cord clamping. Although they did not find any differences in outcomes or Hgb levels, the infants treated with DCC had a *lower C/S* rate (58 versus 78%) (13). In 46 preterm infants 24–32 weeks GA randomized to DCC or ICC, Aladangady et al. reported lower circulating red cell volume with DCC in neonates born by C/S compared with vaginal delivery (15). Because a greater number of infants undergoing DCC were delivered by vaginal delivery, the lower proportion of C/S in this group may have reduced the difference seen between the two approaches.

**Figure 2:**
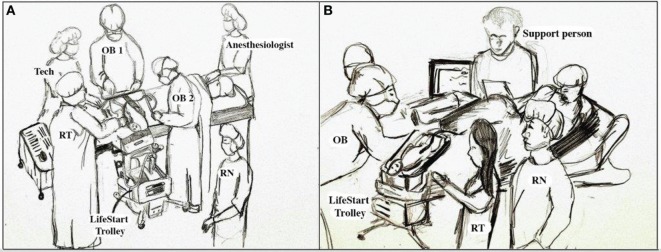
**Personnel and equipment arrangement for neonatal resuscitation with an intact umbilical cord**. **(A)** Operating room and **(B)** labor and delivery suite.

Neonatal resuscitation staff must be aware of all of the procedures in their operating rooms that are utilized to assure the maintenance of the sterile environment when working close to the surgical field. These can include proper scrubbing, gowning, and gloving, as well as the equipment and surfaces that are sterile. Training should be provided by the operating room staff to assure there is confidence that the neonatal team is competent and that they will follow all of the established procedures. Sterile ventilation masks and circuits are not yet available commercially but some vendors are willing to provide sterile equipment for clinical trials at this time. The Lifestart™ bed is lowered to at least the level of the uterine incision and the neonatal provider in sterile attire will provide support as needed (64) (Figure 2A). An additional resuscitation team member should be in the operating room to provide assistance as needed. Thermo-conservation is especially challenging in the operating room, as the temperature tends to be cooler than in other environments. For infants <28 weeks gestation, we used a combination of a chemical mattress on the surface of the Lifestart™ bed, a Mayo stand cover for a sterile water proof barrier, warm sterile towels, and then a sterile polyethylene wrap to cover the infant. When the infant’s umbilical cord was too short to reach the surface of the bed these items were placed directly on the mother. For a vaginal delivery, the Lifestart™ bed is set up and staged near the foot of the bed opposite the OB and brought forward at the level of the mother’s introitus at time of delivery (Figure 2B). This allows the OB to simply set the infant right on the surface of the bed immediately after birth. The Lifestart™ bed is not suitable for transport of the neonate after resuscitation.

### Resuscitation Team Concerns

Usually a baby who requires resuscitation will have the umbilical cord immediately cut and the infant will be removed to a remote location to a radiant warmer. This arrangement provides some advantages to the neonatal team. All of their equipment is readily available in a familiar setup. They should have adequate lighting and a secure, warm, flat platform for the child. They often have some physical separation from the family, which allows them to focus on the neonate. Clinicians also have satisfactory access to the baby as well as an adequate space for the team to work. In a survey of caregivers utilizing the Lifestart™ bed for 78 infants, 24–41 weeks gestational age during DCC, about 12% felt the new arrangement was worse, 61% the same, and 25% better or much better for the clinician (65). The ease of access to the baby and the resuscitation equipment were the primary concerns while using the Lifestart™ bed. In 30% of the deliveries, the length of the umbilical cord was too short to allow the baby to reach the trolley platform. However, the authors’ felt that this number decreased as their skill increased with maneuvering the Lifestart™ bed into position. Interventions successfully provided on the Lifestart™ bed included mask ventilation, endotracheal intubation, surfactant administration, cardiopulmonary resuscitation, and umbilical venous catheterization with intravenous drug administration. The authors concluded that the Lifestart™ bed could be safely and effectively used for resuscitation at both vaginal and C/S births. No adverse events were reported as a result of utilizing the trolley. In a feasibility trial by Yoxall et al. to evaluate whether the Lifestart™ bed could be utilized for a future delayed cord clamping trial, 20 clinicians were interviewed to determine their views on providing resuscitation at birth beside the mother (66). An insufficient cord length to place the baby on the trolley was mentioned by seven clinicians. The majority (10) stated they had no preference between the Lifestart™ bed and the standard equipment, while 5 preferred the standard equipment and 3 preferred to use the trolley. The primary disadvantage of the trolley was felt to be a smaller surface, inadequate space to lie out equipment and concern about the safety of the trolley for the child. Some clinicians may have reservations about the potential to be uncomfortable performing procedures in such close proximity to the family. Of the 16 clinicians who commented on this topic in the study by Yoxall et al., most did not report being uncomfortable themselves but 5 felt that inexperienced clinical staff may be more likely to feel uneasiness.

### Parental Benefits

Parents prefer delayed cord clamping, which has received a lot of media attention and is discussed widely on social media (10). Very little is known about parents’ feelings about witnessing the resuscitation of their newborn during delayed cord clamping. Traditionally, infants who require resuscitation are removed to a remote location away from the mother’s bedside. Often the caregiver’s backs are blocking any view of the child and the clinicians are communicating with each other and no one is communicating with the family. This can create a lot of anxiety for the father who may be worried for both the mother and the child, does not know what is happening, and feels they are not allowed to be with their child (67). Father’s reported feeling stressed, worried, and scared during resuscitation in traditional arrangements. European resuscitation guidelines encourage communication with parents before, during, and after the event as well encouraging parents to touch or hold the baby as soon as possible (68). Resuscitation with an intact cord brings the baby and neonatal care team close to the mother and increases the opportunity for interaction between the baby, the family, and the resuscitation team. Yoxall et al. reported that during cesarean section parents were not able to see due to the screen and where the Lifestart™ bed is stationed (66). Parental interaction may be more pronounced for vaginally delivered infants. Thomas et al. reported that during resuscitation with the Lifestart™ bed the ease of communication and the overall experience for the family was rated as better or much better by two-thirds of clinicians. Some mothers were able to touch their babies and many appreciated being able to see their babies during airway management including endotracheal intubation (65). In the Yoxall et al. feasibility study of using the Lifestart™ bed, 18 clinicians mentioned that parents were able to see and touch their baby during bedside resuscitation and were aware of what was happening with their child (66).

## Risks

Delaying clamping of the cord in preterm infants is well tolerated, and the majority will establish respirations within at least 30 s (35, 64). It appears that for non-breathing infants, a delay in cord clamping may have adverse outcomes of CLD or severe IVH but not death; however, larger trials are needed to confirm this. Two points to consider are, UCM may be a more efficient and time-sensitive technique than a 60-s delay (29), and providing tactile stimulation may be as good as providing PPV in initiating respirations in the newborn (64).

## Conclusion

A placental transfusion with DCC or UCM is an important element in providing a smooth transition for the newborn to extrauterine life. DCC and UCM can enhance arterial oxygen content, hemodynamic stability, and be easily provided in a low-resource setting. Factors including cord clamping time, uterine contractions, umbilical blood flow, respirations, and gravity play an important role in determining placental transfusion volumes. Premature and term newborns who require resuscitation may be the most in need of the benefits of a placental transfusion. While it is possible to provide resuscitation during delayed cord clamping, there are a number of logisitical challenges particularly in the sterile operative field and in premature infants. UCM has not been recommended by any governing body, but may be the most efficient methods to provide a placental transfusion in infants who require resuscitation. Further studies will be required before this practice can come standard of care.

## Author Contributions

AK contributed to the drafting, revision, and final approval of the manuscript. MB contributed to the drafting, revision, and final approval of the manuscript. WR contributed to the drafting, revision, and final approval of the manuscript. KA contributed to the drafting, revision, and final approval of the manuscript.

## Conflict of Interest Statement

The authors declare that the research was conducted in the absence of any commercial or financial relationships that could be construed as a potential conflict of interest.
